# Communicating the move to individualized donor selection policy: Framing messages focused on recipients and safety

**DOI:** 10.1111/trf.17175

**Published:** 2022-11-09

**Authors:** Eamonn Ferguson, Sarah Bowen, Claire Lawrence, Chris Starmer, Abigail Barr, Katy Davison, Claire Reynolds, Susan R Brailsford

**Affiliations:** ^1^ School of Psychology University of Nottingham Nottingham UK; ^2^ National Institute for Health and Care Research Blood and Transplant Research Unit in Donor Health and Behaviour, Department of Public Health and Primary Care University of Cambridge Cambridge UK; ^3^ School of Economics University of Nottingham Nottingham UK; ^4^ Behavioural Practice, KPUK Westminster, London UK; ^5^ LawrencePsychAdvisory Nottingham UK; ^6^ NHS Blood and Transplant/UK Health Security Agency Epidemiology Unit UK Health Security Agency London London UK; ^7^ NHS Blood and Transplant/UK Health Security Agency Epidemiology Unit NHS Blood and Transplant London UK

**Keywords:** behavior, framing, individualized, risk, safety, screening

## Abstract

**Background:**

Men‐who‐have‐sex‐with‐men (MSM) have been deferred from donating blood. However, recent evidence supports the adoption of donor screening based on individuals' sexual behavior over population‐based criteria. We explore how best to frame communications about adopting this change to minimize any potential negative consequences (e.g., reduced donor numbers). We examine the effectiveness of risk (emphasizing safety vs. emphasizing low risk), and focus (donor vs. recipient) frames on intentions to donate blood (approach) or feeling deterred from donating (avoid), and mechanisms linked to under‐reporting sexual behavior.

**Study Design and Methods:**

We conducted a 2 (risk frame: risk vs. safety) by 3 (focus: donor vs. recipient vs. both) between‐subjects online experiment (*n* = 2677). The main outcomes were intentions to donate and feelings of being put‐off/deterred from donating (both for self and others). We also assessed the extent that forgetting, embarrassment/shame, and question irrelevance were perceived to be associated with under‐reporting sexual behavior.

**Results:**

Frames that focused on safety or a recipient resulted in people reporting being less deterred from donating. Regardless of frame, people from ethnic minorities were more likely to feel deterred. Embarrassment/shame followed by forgetting and perceived irrelevance were the main reasons for under‐reporting sexual behaviors, especially in ethnic minorities, and smartphones were perceived as an acceptable memory aid for sexual behavior.

**Discussion:**

Blood services moving to an individualized policy should frame donor selection in terms of safety and/or a recipient focus, explore sensitivities in ethnic minority communities, consider ways to normalize reporting sexual behavior, and use smartphones as a memory aid.

## 1 INTRODUCTION

Internationally, blood services have adopted population‐based screening policies for men‐who‐have‐sex‐with‐men (MSM), resulting in either permanent or time‐based deferrals, usually between 3 and 12 months since the last sex with another man.^1^, ^2^, ^3^ However, accumulated evidence^4^, ^5^, ^6^, ^7^ and improved Nucleic Acid Testing^8^ indicate that such policies require review to ensure that they are justifiable, fair, and equitable.^9^, ^10^, ^11^ Instead, deferring all donors engaging in high‐risk sexual behavior has been recommended.^12^, ^13^, ^14^ In 2020, the FAIR (For the Assessment of Individual Risk) project recommended that the United Kingdom (UK) blood services replace time‐based MSM deferrals with an individualized assessment of all donors based on sexual behavior and sexually transmitted infection history.^15^ This paper explores the resulting challenge of how best to frame communications about such a policy change to minimize potential negative consequences (e.g., putting people off donating) within an approach‐avoidance framework.

### 1.1 Approach‐avoidance framework

The approach‐avoidance distinction refers to a fundamental mechanism underlying human motivation.^16^, ^17^, ^18^, ^19^, ^20^, ^21^, ^22^, ^23^, ^24^, ^25^, ^26^ This distinction proposes that motivation is driven by two systems. The first encourages behaviors that move the person toward stimuli (goals) that they deem beneficial, and the second inhibits movement toward potentially harmful stimuli.^19^, ^20^, ^21^, ^22^, ^23^, ^24^, ^25^, ^26^ The distinction is supported by evidence that these two systems (i) have separate neurological substrates^16^, ^21^, ^23^, ^24^, ^25^, ^26^ and (ii) are conserved across species.^19^ There is also evidence that people intuitively evaluate stimuli in this way, with positively evaluated stimuli more likely to be approached and negatively evaluated stimuli more likely to be avoided.^19^ However, in many contexts, evaluations of stimuli are mixed, and it is how the decision‐maker balances approach and avoidance tendencies that drives behavior.^16^, ^22^ Blood donation presents a mixed evaluative context within which donors weigh up positive (e.g., warm‐glow experienced from donating associated with approach) and negative (e.g., fear of fainting associated with avoidance) attributes.^27^ The framing of an individualized blood donor screening policy based on donors' sexual behavior, might alter the balance between approach and avoidance.^19^, ^27^ We explore the balance between approach (intentions‐to‐donate) and avoidance (being put‐off donating) to establish the best way to frame communications to minimize avoidance or increase approach decisions (Supplementary File S1 for more detail).

### 1.2 Framing the move to individualized screening approach: Risk and altruism

#### 1.2.1 Framing risk

Under the precautionary principle or a risk management approach, any policy change must not increase potential harm.^28^, ^29^, ^30^, ^31^ Here, potential harm refers to the risk (with probability r) that an infectious donation is made within a non‐detectable window period (viral residual risk). Therefore, the expected value of a transfusion of donated blood is $\left(1-r\right)G+\mathrm{rB}$, where 0<r<1, $\left(G\right)$ is the net benefit generated by a transfusion received safely, and $\left(B\right)$ is the net cost generated by a transfusion leading to infection. Under the precautionary principle, the change to individualized screening must reduce, or at least not increase, viral residual risk ($r^{\prime }\leq r$, where $r^{\prime }$ is the probability of an infectious donation after the change). A primary behavioral concern of transfusion services is that such a policy does not put‐off donors. Therefore, we investigate whether, to increase approach/minimize avoidance decisions to donate, communications should frame the policy change $(r^{\prime }\leq r$) in terms of *increased recipient safety* ($\left(1-r^{\prime }\right)G\geq \left(1-r\right)G$) or *reduced recipient risk* ($r^{\prime }B\leq \mathrm{rB}$).

Theory and evidence show that losses loom larger than equivalent gains, implying that focusing on reducing risk will be more effective than enhancing perceived safety.^32^, ^33^ Historically, blood services have focused on objective risk.^34^ However, peoples' responses to risk are influenced by heuristics and emotions.^35^, ^36^ For example, messages and context highlighting donation/transfusion risks are associated with perceptions of reduced safety of blood.^37^, ^38^ Thus, frames focused on risk may induce avoidance. In terms of emotions, the risk‐as‐feelings hypothesis^35^ and the affect heuristic^36^ suggest that, for positive events, communicating increased safety (gains) will enhance the perceived benefits, and subsequent approach behavior.^19^, ^22^ As evidence suggests the FAIR policy is viewed positively, frames emphasizing safety should be more effective than those emphasizing minimizing risk.^15^

#### 1.2.2 Other‐regarding frames

Frames that have an other‐regarding focus, where the person acts to benefit the well‐being of others, have been shown to increase cooperative health behaviors.^39^, ^40^, ^41^, ^42^, ^43^, ^44^, ^45^ Therefore, frames focusing on the recipient of blood should motivate approach decisions. Hence, we expect recipient‐focused and safety‐frames to be more effective than donor‐focused and risk‐frames in motivating decisions increasing approach/minimizing avoidance.

### 1.3 Reporting sexual behavior: approach‐avoidance mechanisms

While ensuring a policy change does not put‐off donors is a primary consideration, a second is ensuring those attending comply with the new selection criteria.^12^, ^46^, ^47^ Thus, we also explore whether potential donors are aware of factors that may influence non‐compliance. We examine awareness of three factors that may influence non‐compliance: (i) anticipated shame/embarrassment of being asked about sexual behaviors,^48^, ^49^, ^50^ (ii) forgetting sexual behaviors,^51^ and (iii) the perception that the questions are irrelevant because blood is tested for infections.^52^ Understanding awareness of non‐compliance mechanisms will inform targeted strategies to reduce non‐compliance.

## 2 METHODS

### 2.1 Sampling procedure

**FIGURE 1:**
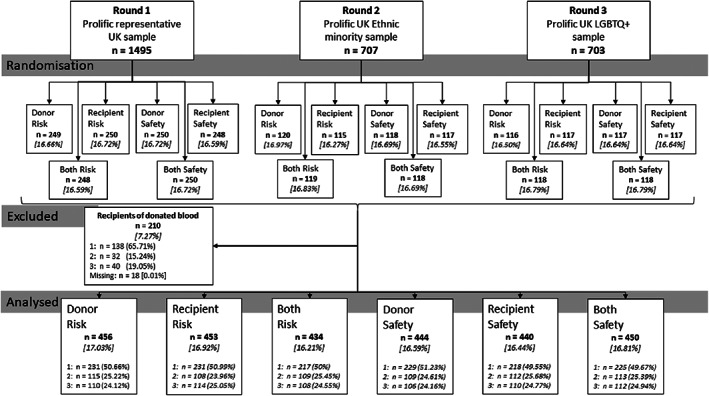
Sampling strategy (1, 2 & 3 = sampling rounds 1, 2 & 3 respectively)

Stratified random sampling was employed (Figure 1), through Prolific (https://www.prolific.co/about/), to oversample LGBTQ+ and ethnic minority communities. Initially, a representative sample (age, gender, and ethnicity) of the UK population (*n* = 1495) was recruited, followed by additional samples of UK participants exclusively from ethnic minorities (*n* = 707) and LGBTQ+ (*n* = 703) communities. All data were collected in February 2021.

### 2.2 Design

**FIGURE 2:**
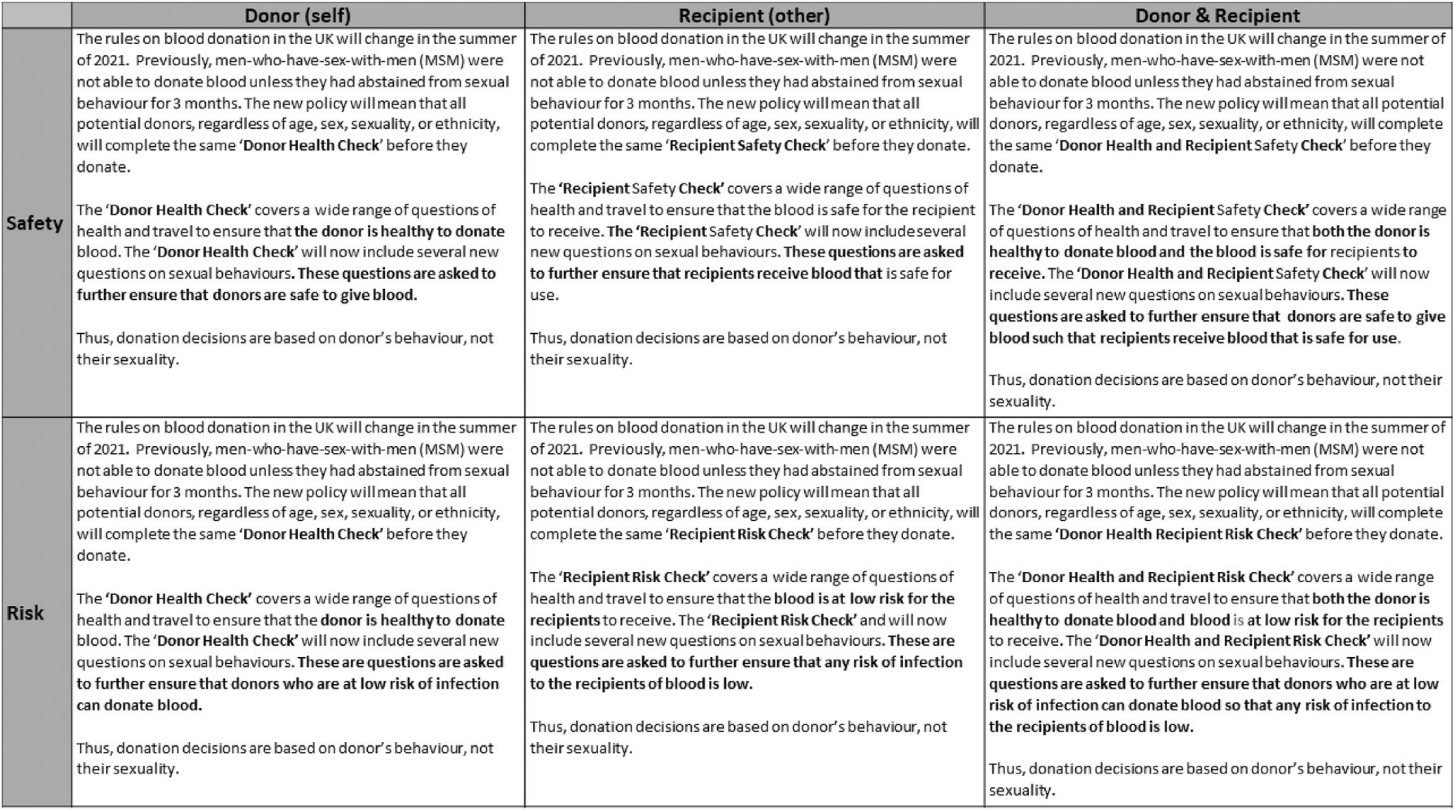
Risk frames (risk vs. safety) by focus (donor, recipient or both)

Participants were randomly assigned to one of the six conditions formed by crossing 2 *risk* (risk vs. safety) with 3 *focus* (donor, recipient or both) *frames* (Figure 2: Supplementary File S2 for more details).

## 3 MEASURES

### 3.1 Pre‐manipulation measures

#### 3.1.1 Demographics

We recorded age, gender identity (Female/Male/Gender non‐conforming/Other/Prefer not to say), ethnicity across the 18 UK Office of National Statistics categories and sexual identity (Supplementary File S3).

#### 3.1.2 Blood donation history

Participants were asked whether they had ever donated blood, and if yes, whether this was in the UK, and the time since their last donation (less than a month ago/2 to 12 months ago/12 months to 2 years ago/longer than 2 years ago/cannot remember). Respondents were coded as non‐donors, lapsed (donors who had not donated in the last 2 years), and current (donors who had donated within the last 2 years) donors. Participants were asked if they had ever been a recipient of blood or its components (Yes/No).

### 3.2 Post‐manipulation measures

After reading the communication participants had been assigned to, participants answered the following questions.

### 3.3 Manipulation check

We assessed the *focus* (“Who is the focus of the statement?” 0 = the donor through 5 = both equally, to 10 = recipients), and *salience* (“To what extent does the statement make you think about the patients who receive blood?” 1 = Not at all, to 7 = Completely) of the communications.

### 3.4 Main outcomes

The main outcomes are described below.

#### 3.4.1 Approach and avoidance

*Approach* was assessed by the sum of two yes/no intentions items: (i) Do you plan to donate blood in the near future? and (ii) Would you be willing to donate blood? *Avoidance* was assessed using two items: (i) To what extent would the statement *put you off* donating blood? (*Self‐Deter*) and (ii) To what extent do you think the statement would *put others off* donating blood? (*Other‐Deter*) (from 1 = Not at all to 7 = Completely). These indices of approach and avoidance were normalized between 0 and 1 (Supplementary File S1 for details and full rationale).

#### 3.4.2 Approach‐avoidance balance index (AABI)

The relative balance of approach versus avoidance was assessed with an approach‐avoidance balance index (AABI) ranging from −1, strong motivation toward avoidance, 0, equal approach and avoidance, and + 1, strong motivation toward approach. Two AABIs were constructed: (i) *Self‐AABI* based on the normalized approach index minus the normalized self‐deter index and (ii) *Normative‐AABI* based on the normalized approach index minus the normalized sum of the self‐deter and other‐deter indices. Negative conditional cooperation indicates people are less likely to act if they think others will not act.^53^ Consistent with this there was a strong association between self‐ and other‐deter (*r =* 0.508, *p <* 0.001) (Supplementary File S1 for formulae and more details).

#### 3.4.3 Mechanisms of non‐compliance

All participants saw the same stem – “To what extent do you think each of the following factors influences how accurately people report on their sexual behavior over the last 3 months?” (1 = Not at all, 7 = Completely): (i) they had *forgotten* aspects of their previous sexual behavior, (ii) feeling *embarrassed* to report on their sexual behavior, (iii) feeling *ashamed* to report on their sexual behavior and (iv) feeling that the questions are *not relevant* as all blood is tested anyway and so decide not to report their sexual behavior. A *negative emotions* score was calculated as the average response of feeling embarrassed and ashamed (*r* = 0.794, *p* < 0.001).

### 3.5 Secondary outcomes

We assessed perceived “safety” of blood, perceived “fairness” of the policy, and use of a smartphone to aid recall of sexual behavior (Supplementary File S4 for details on measures and scoring).

### 3.6 Statistical analysis strategy

All analyses were conducted in Stata 17 and SPSS 27. All *p*‐values are two‐tailed. Seven percent (*n* = 210) of the sample reported that they had received blood and were excluded from the analysis. The results were not sensitive to the exclusion of recipients.

## 4 RESULTS

### 4.1 Sample characteristics

Sample characteristics are summarized in Table 1. For the regression analysis, a single category, LGBQ+, was created encompassing Lesbian, Gay, Bisexual, Queer, Pansexual, Bi‐curious, and Asexual: *n* = 788. Balance tests confirm randomization (Supplementary File S5).

**TABLE 1:** Analyzed sample characteristics.

	*N*	%	Mean	Proportion				
			Age	Men	Asian	Black	Mixed	White
Blood donation history								
Non‐donor	1755	65.56	34.74	0.40	0.09	0.19	0.08	0.62
Lapsed donor	600	22.41	47.44	0.44	0.04	0.11	0.04	0.78
Current donor	315	11.77	36.43	0.43	0.08	0.09	0.05	0.76
Prefer not to say	7	0.26	44.43	0.43	0.13	0.25	0.13	0.31
Self identified sexual orientation								
Asexual	50	1.87	35.06	0.32	0.04	0.12	‐	0.84
Bisexual	366	13.67	29.26	0.21	0.07	0.06	0.02	0.83
Gay	118	4.41	36.14	0.91	0.07	0.05	0.01	0.87
Heterosexual/straight	1882	70.30	40.63	0.46	0.08	0.20	0.08	0.61
Lesbian	93	3.47	30.68	‐	0.05	‐	0.01	0.94
Queer	31	1.16	29.52	0.13	0.03	0.06	0.03	0.87
Pansexual	48	1.79	26.06	0.17	0.06	0.08	0.04	0.79
Bi‐curious	31	1.16	27.23	0.32	0.16	0.16	0.06	0.55
Prefer not to say	58	2.17	35.55	0.24	0.16	0.14	0.04	0.63
Total	2677	100	37.80	0.42	0.16	0.07	0.08	0.68

*Note*: Non‐donor: Never donated; Lapsed: Donated more than 2 years ago; Current: Donated within the last 2 years. Asexual: People who self‐identify as asexual; Bisexual: People who self‐identify as bisexual; Gay: People who self‐identify as gay; Straight: People who self‐identify as straight; Lesbian: People who self‐identify as lesbian; Queer: People who self‐identify as queer; Pansexual: People who self‐identify as pansexual; Bi‐curious: People who self‐identify as bi‐curious.

Examining Table 2, intentions to donate blood were high (*M = 1.42, SD = 0.68, Normalized mean = 0.71*), and self (*M = 1.99, SD = 1.46, Normalized mean = 0.17*) and other‐avoidance (*M = 3.01, SD = 1.58, Normalized mean = 0.33*) were low. AABI scores were positive indicating feelings of approach toward donation dominated (*Self‐AABI: M = 0.55, SD = 0.45; Normative‐AABI: M = 0.46, SD = 0.43*). Perceived safety of blood (*M = 11.21, SD = 2.68*) and fairness of the policy (*M = 24.27, SD = 3.30*) were high and significantly associated with greater approach, and lower avoidance. Anticipated negative emotions were the most likely to be seen to influence the under‐reporting of sexual behavior (*M = 5.36, SD = 1.23*), followed by the perceived irrelevance of questions (*M = 4.98, SD = 1.57*), and forgetting (*M = 4.03, SD = 1.58*). Awareness of all three mechanisms were weakly positively correlated with both self‐ and other‐avoidance. Greater awareness of forgetting was positively associated with the appreciation of smartphones as a memory aid.

**TABLE 2:** Matrix summarizing outcome variable sample means, standard deviations, and pairwise correlation coefficients with significance

	M	SD	1.	2.	3.	4.	5.	6.	7.	8.	9.	10.
1. Approach (Intentions)	1.42	0.68	1.00									
2. Self‐avoidance (Self‐Deter)	1.99	1.46	−0.17***	1.00								
3. Other‐avoidance (Other‐deter)	3.01	1.58	−0.07***	0.51***	1.00							
4. Normative‐AABI	0.46	0.43	0.86***	−0.57***	−0.50***	1.00						
5. Safety	11.21	2.68	0.24***	−0.36***	−0.19***	0.35***	1.00					
6. Fairness	24.27	3.30	0.13***	−0.39***	−0.33***	0.30***	0.44***	1.00				
7. Forgetting	4.03	1.58	0.01	0.10***	0.11***	−0.05**	−0.08***	−0.00	1.00			
8. Mean anticipated negative emotion	5.36	1.23	−0.02	0.08***	0.13***	−0.08***	−0.16***	−0.06*	0.24***	1.00		
9. Perceived Irrelevance	4.98	1.57	−0.05**	0.11***	0.11***	−0.10***	−0.16***	−0.14***	0.19***	0.42***	1.00	
10. Effectiveness of smartphones	3.49	1.74	0.11***	0.00	0.01	0.09***	0.13***	0.14***	0.10***	−0.08***	−0.12***	1.00

*Note*: *p < 0.05, **p < 0.01, ***p < 0.001. Approach and self‐ and other‐avoidance are raw scores. Approach is the sum of the two intention items (range 0–2). Normative‐AABI is normalized (0–1). Safety (range 2–14). Fairness (range 4–28). Forgetting: Anticipated forgetting on the under‐reporting of sexual behavior (range 1–7). Anticipated negative emotions (shame and embarrassment) on under‐reporting sexual behavior (range 1–7). Anticipated irrelevance of questions on the under‐report sexual behavior (range 1–7). Effectiveness of smartphones: The extent to which people feel asking people to use their mobile phone to aid recall over the last 3‐months would be an effective strategy to increase compliance (range 1–7).

### 4.2 Manipulation checks for effects of frame and focus

The perceived patient focus was higher in the recipient frames, particularly for the combined donor‐recipient frame (Supplementary File S6); supporting the validity of the recipient frames.

### 4.3 Frames and approach‐avoidance considerations

Analysis of normalized‐approach (Table 3, Model 1) indicates no framing effects. Higher normalized‐approach was observed for lapsed and current donors and younger participants. The finding that LGBQ+ people were less likely to approach is likely a negative suppressor effect due to age as the zero‐order relationship between LGBTQ+ and approach is positive (Supplementary File S7).

**TABLE 3:** OLS regression for normative intentions, normative‐self‐deferral, normative‐other‐deferral, normative self‐other deferral, self‐approach‐avoidance score, and approach‐avoidance score.

	(1)		(2)		(3)		(4)		(5)		(6)		
	Normalized approach		Normalized self‐avoidance		Normalized other‐avoidance		Normalized normative‐avoidance		Self‐AABI		Normative‐AABI		
	β	CI [95%]	β	CI [95%]	β	CI [95%]	β	CI [95%]	β	CI [95%]	β	CI [95%]
Risk frame												
Safety	0.003	[−0.040,0.045]	−0.044**	[−0.075, −0.012]	−0.048**	[−0.083, −0.013]	−0.046**	[−0.074, −0.017]	0.046	[−0.011,0.103]	0.049	[−0.006,0.103]
Other‐regarding frames												
Recipient	0.023	[−0.020,0.065]	−0.037*	[−0.068, −0.006]	−0.035*	[−0.070, −0.001]	−0.036*	[−0.064, −0.008]	0.059*	[0.003,0.116]	0.058*	[0.004,0.112]
Both	0.011	[−0.032,0.054]	−0.021	[−0.052,0.011]	−0.025	[−0.060,0.010]	−0.023	[−0.051,0.006]	0.032	[−0.025,0.090]	0.034	[−0.021,0.089]
Risk × other‐regarding frames												
Safety × Recipient	0.000	[−0.060,0.061]	0.047*	[0.003,0.092]	0.068**	[0.019,0.117]	0.058**	[0.017,0.098]	−0.047	[−0.128,0.034]	−0.057	[−0.134,0.021]
Safety × Both	−0.023	[−0.083,0.038]	0.034	[−0.011,0.078]	0.049	[−0.000,0.099]	0.041*	[0.001,0.082]	−0.056	[−0.137,0.025]	−0.064	[−0.142,0.014]
Age	−0.007***	[−0.008, −0.006]	0.000	[−0.000,0.001]	0.000	[−0.001,0.001]	0.000	[−0.001,0.001]	−0.007***	[−0.008, −0.006]	−0.007***	[−0.008, −0.005]
Men	−0.016	[−0.041,0.009]	0.042***	[0.023,0.061]	0.011	[−0.010,0.031]	0.026**	[0.009,0.043]	−0.058***	[−0.092, −0.024]	−0.042*	[−0.075, −0.010]
LGBQ+	−0.037*	[−0.067, −0.006]	−0.025*	[−0.048, −0.003]	−0.026*	[−0.051, −0.001]	−0.026*	[−0.046, −0.005]	−0.011	[−0.052,0.030]	−0.01	[−0.050,0.029]
Ethnicity												
Asian	−0.02	[−0.057,0.016]	0.129***	[0.102,0.157]	0.084***	[0.054,0.114]	0.107***	[0.082,0.132]	−0.150***	[−0.199, −0.100]	−0.127***	[−0.174, −0.080]
Black	0.003	[−0.048,0.055]	0.082***	[0.044,0.120]	0.062**	[0.020,0.105]	0.072***	[0.037,0.107]	−0.078*	[−0.148, −0.008]	−0.068*	[−0.135, −0.002]
Mixed	−0.053*	[−0.100, −0.005]	0.061***	[0.027,0.096]	0.011	[−0.027,0.050]	0.036*	[0.005,0.068]	−0.114***	[−0.177, −0.050]	−0.088**	[−0.149, −0.028]
Donor status												
Lapsed donor	0.137***	[0.105,0.169]	−0.069***	[−0.093, −0.046]	−0.034*	[−0.060, −0.007]	−0.052***	[−0.073, −0.030]	0.207***	[0.164,0.250]	0.189***	[0.148,0.230]
Current donor	0.301***	[0.262,0.340]	−0.043**	[−0.072, −0.014]	−0.003	[−0.035,0.029]	−0.023	[−0.049,0.003]	0.344***	[0.292,0.397]	0.324***	[0.274,0.375]
Constant	0.003	[−0.040,0.045]	−0.044**	[−0.075, −0.012]	−0.048**	[−0.083, −0.013]	0.256***	[0.219,0.292]	0.756***	[0.682,0.829]	0.656** *	[0.586,0.727]
$R^{2}$	0.145		0.079		0.029		0.064		0.121		0.115	
*N*	2552		2552		2552		2551		2551		2550	

*Note*: *p < 0.05, **p < 0.01, ***p < 0.001. Frames: Risk frame is the comparison condition; Focus Frames: Donor frame is the comparison condition; Ethnicity: People from white ethnic backgrounds act as the comparison community, Donor status: non‐donors are the comparison group; Self‐AABI: Approach‐avoidance index based on normalized approach minus normalized self‐avoidance; Normative‐AABI: Approach‐avoidance index based on normalized approach minus normalized self plus other avoidance. Coefficients are unstandardized.

Exposure to a safety‐frame, compared to a risk‐frame, or a recipient‐frame compared to a donor‐frame reduced (i) Self‐Avoidance (Model 2), (ii) Other‐Avoidance (Model 3), and (iii) Normative‐Avoidance (Model 4). There was also a significant interaction between the risk‐ and focus‐frames on Self‐Avoidance (Model 2), Other‐Avoidance (Model 3), and Normative‐Avoidance (Model 4). Examining the margins for these interactions indicates that the highest Avoidance occurred for a combination of risk‐ and donor‐frames (Supplementary File S8).

People from Asian, Black, and Mixed ethnic communities were more likely to be deterred from donating relative to people from White communities and people from LGBTQ+ communities less deterred compared to straight people. Both lapsed and current donors were less likely to be deterred compared to non‐donors (Model 2). The demographic effects on Other‐Avoidance (Model 3) are similar to Self‐Avoidance (Model 2), except for gender, Mixed ethnicity, and being a current donor. Analysis of *Self‐AABI* and *Normative‐AABI* scores (Models 5 & 6) indicate that exposure to a recipient‐frame, compared to a donor‐frame, reduced avoidance relative to approach.

### 4.4 Awareness of mechanisms of non‐compliance

There were no framing effects on the awareness mechanisms (Supplementary File S9). However, relative to women, men reported less awareness of all mechanisms, younger respondents reported more awareness of forgetting and negative emotions, and current donors reported less awareness of negative emotions as mechanisms leading to under‐reporting.

Relative to those from White communities, people from Black communities reported more awareness of all three mechanisms, and people from Asian communities reported more awareness of forgetting.

### 4.5 Effects of frames on perceived safety and fairness

There were no significant framing effects on perceived safety or fairness. However, perceived safety was lower among older, Asian, Black, and Mixed ethnicity participants and higher among lapsed and current donors and people from LGBQ+ communities (Supplementary File S10).

## 5 DISCUSSION

Blood services do not want to lose donors due to a policy change. The main finding of this paper shows that frames focusing on increasing safety (rather than reducing risk) and/or the recipient (rather than the donor) decrease participants' likelihood of being put‐off donating following a policy change involving individualized risk assessment of donors' sexual behavior and infection history. These findings add to the growing body of evidence that other‐regarding frames, emphasizing the benefits to others, enhance health‐based cooperation^39^, ^40^ and that safety‐frames are effective when a policy change is viewed positively.^15^ Also, consistent with the idea that expectations about what others guide personal behavior by providing a normative justification (*I am doing what others would do*),^53^ we find that exposure to recipient‐focused and safety‐based frames also reducing expectations that others would be deterred.

We also observed that current donors, lapsed donors, and LGBQ+ participants reported lower avoidance. Thus, the number of current active donors should not reduce under this policy. However, people from Asian, Black, and Mixed ethnic communities were more likely to be deterred. People from ethnic minority communities are, in general, less likely to donate,^54^, ^55^, ^56^, ^57^ so it is of concern that this type of policy change is linked to greater avoidance in these communities.

We found that people were aware of the mechanisms linked to under‐reporting of sexual behavior: (i) feeling embarrassed,^48^ (ii) forgetting^51^ or (iii) questions perceived as irrelevant.^52^ Embarrassment/shame was rated the most likely mechanism leading to inaccurate reporting, followed by irrelevance, and forgetting. The use of smartphones as aide‐memoires was highlighted as a potentially effective strategy to enhance accurate recall. Awareness was not influenced by frames but people from Black or Asian ethnic minority communities were more likely to report greater awareness.

## 6 IMPLICATIONS FOR BLOOD SERVICES AND RESEARCH

As more blood services adopt individualized approaches on sexual behavior and sexual health, an implication from this research, is to consider framing communications to focus both on safety and recipients.^58^ The findings also indicate useful directions for future research on individualized policies. First, we need to know why people from ethnic minority communities are more likely to indicate that they would be deterred from donating under such a policy. This would help us understand the relevant cultural variations which might be driving this and identify strategies to mitigate concerns and encourage donations, thereby increasing donor pool diversity and improving donor‐recipient matching for effective treatments.^55^, ^56^, ^57^ Second, while shame/embarrassment was reported as the main reason for not reporting sexual behavior, it is important to acknowledge that in this context this likely reflects everyday emotional reactions rather than clinical presentations of shame/embarrassment.^49^, ^59^, ^60^ As such, practical steps to minimize embarrassment through for example increased privacy to complete the screening questions should be explored.^59^ Third, the importance of smartphones as memory aids, to enhance compliance should be examined. Initially, objective effectiveness could be explored. For example, if people are asked to recall their sexual behavior without their phone and again with their phone (as a means to reconstruct events and dates), are they able to recall more information about the number and nature of sexual encounters after using their phone? If so, the use of smartphones should be trialed in terms of acceptability and feasibility from donors and staff before any procedural roll‐out.

## FUNDING INFORMATION

This work was funded by a grant from the UKFORUM to Eamonn Ferguson & Claire Lawrence and by the Economic and Social Research Council [grant number ES/P008976/1] to Chris Starmer. The views expressed in this paper are those of the authors and do not reflect any of the organizations or funders related to this paper.

## CONFLICT OF INTEREST

The authors have disclosed no conflicts of interest.

## Supporting information


**Appendix S1.** Supporting Information.
