# Supplementary material for: Communicating the move to individualized donor selection policy: Framing messages focused on recipients and safety
Source: Transfusion. 2022 Nov 9;63(1):171–81. doi: 10.1111/trf.17175 (PMC10099824; doi:10.1111/trf.17175)
Supplement: Supplementary file 1 — Appendix S1. Supporting Information. [file TRF-63-171-s001.docx]

**Supplementary Files for “****Communicating the move to individualized donor screening: Framing messages based on recipients and safety.”**

Table of Contents

[Supplementary File S1: Approach-Avoidance Distinction and Operationalizing Approach-Avoidance in the Context of Blood Donor Decision Making 2](#_Toc118717186)

[**Conceptualizing Approach-Avoidance for Blood Donor Motivations** 2](#_Toc118717187)

[**Normalizing Individual Indices of Approach and Avoidance** 6](#_Toc118717188)

[**Creating Overall Normalized Approach-Avoidance Balance Indices** 6](#_Toc118717189)

[Supplementary File S2: Structure of the Policy Communication Messages 7](#_Toc118717190)

[Supplementary Files S3: Sample Structure 9](#_Toc118717191)

[**Sampling: Ethnicity and Sexuality Classifications** 9](#_Toc118717192)

[Supplementary File S4: Secondary Outcomes 11](#_Toc118717193)

[**Perceived Safety of Blood** 11](#_Toc118717194)

[**Perceived Fairness and Equality** 11](#_Toc118717195)

[**Potential Mechanisms to Mitigate Non-Compliance** 11](#_Toc118717196)

[Supplementary File S5: Balance tests 12](#_Toc118717197)

[Supplementary File S6: Other Regarding Focused Frames Manipulation Checks 15](#_Toc118717198)

[Supplementary File S7: Negative Suppressor Effect of Age on the LGBTQ+ - Approach Association 17](#_Toc118717199)

[Supplementary File S8: Margins Analysis of Approach-Avoidance Interactions 19](#_Toc118717200)

[Supplementary File S9: Regression Models for Awareness of Mechanisms Linked to the Anticipation of Under-Reporting of Sexual Behaviour 23](#_Toc118717201)

[Supplementary File S10: Safety and Fairness Regression Models 25](#_Toc118717202)

# Supplementary File S1: Approach-Avoidance Distinction and Operationalizing Approach-Avoidance in the Context of Blood Donor Decision Making

## **Conceptualizing Approach-Avoidance for Blood Donor Motivations**

Approach and avoidance are seen as fundamental motivations underlying a wealth of human and other animal behaviors [1-7]. Approach and avoidance are conceptualized as two separate systems [2, 6, 8-11]. The approach system supports behaviors that allows the organism to approach goals that they deem beneficial or desirable. This may be towards appetite stimuli that range from basics such as food, water, or safety through to social rewards [1, 6, 11]. However, key here is that approach does not necessarily cause actions that move an organism towards beneficial behaviors. Rather approach is more simply a motivation that directs action *towards* a goal. That goal or its fulfilment may not necessarily be beneficial for the organism, but the organism perceives it to be beneficial to them. In contrast, the avoidance system acts to inhabit movement towards potentially harmful stimuli that may cause harm to the organism, such as a threat to bodily injury, attack as well as psychological threats [1-7]. This approach-avoidance distinction is supported by evidence that these two systems (i) have separate neurological substrates (e.g., behavioral activation and behavioral inhibition systems; behavioral approach and withdrawal systems) [8-11] and (ii) are conserved across species [1]. Furthermore, people intuitively evaluate stimuli as positive (to be approached) and negative (to be avoided) and this reinforces and guides initial and subsequent behavior [1]. As stated above, it is the perceived benefits and costs, which do not necessarily to equate with objective benefits and costs, which drive behavior.

The approach-avoidance distinction underlies many theories of human behavior [1]. Drawing on the review in [1], we see, for example: (i) Maslow distinguishes ‘deceit needs’ (avoidance) and ‘growth needs’ (approach), (ii) Hull’s drive theory distinguishes two types of drive, conditioned appetite drives (approach) and conditioned aversive drives (avoidance), and (iii) and Gray’s Reinforcement Sensitive Theory (RST) of personality is based around a behavioral activation system (BAS: approach) and a Behavioral Inhibition System (BIS: avoidance), which are have clear separate conceptual nervous systems [6, 10-11]. However, in all cases, the approach-avoidance mechanisms do not work in isolation, independent of context. Rather, activation of approach and avoidance is contingent on the local context, the person’s emotional state, goals, and perceived benefits (costs) associated with fulfilling those goals. Humans tend to afford objects with evaluative meaning (e.g., good or bad), and the way an object is evaluated or appraised by the individual differentially activates the approach or avoidance tendencies [1, 3, 7]. Thus, affording positive or negative attributes of an object will differentially activate approach or avoidance systems. In many cases the situation is not so clear that just one of these systems is activated. Rather it is the relative balance between approach and avoidance that drives behavior and this is determined, in part, by the way the context is appraised or evaluated (good-bad, safe-risky, positive-negative, likely to be satisfying-not satisfying) by the person [1, 3, 7].

Indeed, blood donation is a mixture of positive attributes, such as helping others, the warm-glow of giving, and health checks, as well as negative attributes including anxiety, and fear of fainting. The approach system will be stimulated by the positive attributes and evaluations and the avoidance system by the negative attributes and evaluations. The donor weighs these up and decides to approach (donate) or avoid (not donate) [12]. As approach-avoidance decisions can be influenced by the context, any change that alters the way that blood donation is perceived or evaluated by the potential donor will shift this balance towards approach if this evaluation enhances positive attributes and towards avoidance if it highlights negative attributes. As such, a change in policy to one based on individualized screening on sexual behaviour and sexual history, can alter this balance [12]. Indeed, as the FAIR policy was perceived, overall, as a positive change in terms of lower risk and enhanced equality and fairness, this should afford approach decisions [13].

In the blood donation context, approach decisions can be conceptualized by intentions-to-donate. However, it should be noted that low intent is not the same as actively deciding to avoid donating. Low intent may arise, for example, from inertia or just low approach motivation. Whereas deciding not to donate is an active decision to avoid donating (being deterred from donating). Thus, low intentions are not equivalent to active avoidance. Thus, both approach (intentions-to-donate) and avoidance (being deterred from donating blood) need to be assessed separately. Furthermore, as a final decision to approach or avoid is based on how people weigh up the relative strength of approach and avoidance tendencies, we operationalized the relative balance between approach and avoidance. To achieve this, we normalized scores on approach (intentions-to-donate) and avoidance (being deterred from donating) to be on the same scale of 0-1. We then calculated the difference between these two normalized scores to create an index of the relative balance of approach to avoidance. The specific details of this process is described below in the next section.

**References**

[1]. Elliot, A.J., Covington, M.V. Approach and Avoidance Motivation. Educ Psychol Rev*.* 2001*;* 13: 73–92,

[2]. Corr PJ. Approach and Avoidance Behaviour: Multiple Systems and their Interactions. Emot Rev. 2013;5:285-290.

[3]. Sherman DK, Mann T, Updegraff JA. Approach/Avoidance Motivation, Message Framing, and Health Behavior: Understanding the Congruency Effect. Motiv Emot. 2006;30:165-169.

[4]. Mowrer, 0. H. (1960). Learning theory and behavior. New York: Wiley

[5]. Hull, C. L. (1952). A behavior system. New Haven: Yale University Press

[6]. Gray JA, & McNaughton (2000). The neuropsychology of anxiety: An enquiry into the functions of the septo-hippocampal system (2nd edn). Oxford: Oxford

[7]. Carver CS. Approach, Avoidance, and the Self-Regulation of Affect and Action. Motiv Emot*. 2006;* 30: 105–110.

[8]. Cloninger C. A systematic method for clinical description and classification of personality variants. Arch. Gen. Psychiat. 1987; 44: 573-588

[9]. Depue R, Collins PF. (). Neurobiology of the structure of personality: Dopamine, facilitation of incentive motivation, and extraversion. Behav. Brain Behav. Brain Sci, 1999; 22: 491-569.

[10]. Gray, J. (1982). The Neuropsychology of anxiety, University Presss, New York

[11]. Corr PJ. Reinforcement sensitivity theory and personality. Neurosci Biobehav Rev. 2004; 28: 317-32.

[12]. Ferguson E, Chandler S. A Stage Model of Blood Donor Behaviour: Assessing Volunteer Behaviour. J Health Psychol. 2005; 10: 359–37

[13]. FAIR. Can donor selection policy move from a population based donor selection policy to one based on a more individualized risk assessment? Conclusions from the For the Assessment of Individualized Risk (FAIR) group. 2020. [cited Sep 23, 2021], Available from: <https://nhsbtdbe.blob.core.windows.net/umbraco-assets-corp/21001/fair_sabto_20201211.pdf>

## **Normalizing Individual Indices of Approach and Avoidance**

The indices of Approach and Avoidance were normalized to between 0 and 1 using the following formulae. Where: *maxscore* = maximum possible score on the scale, *minscore* = minimum possible score on the scale, *score_i_* = the individual actual score on the scale.

$$Normalise{d score}_{i}=\frac{score_{i}-minscore}{maxscore-minscore}$$

## **Creating Overall Normalized Approach-Avoidance Balance Indices**

To create balance scores to represent the relative strength of approach versus avoidance we calculated approach-avoidance balance indices (AABI) based on the individual normalized indices. These range from -1 to 1, where a score of -1 represents the strongest force towards avoidance, 0 represents the case when the forces cancel each other out, and +1 presents the strongest force towards approach.

$$Self\_AABI_{i}=Approac{h\_normalised}_{i}-Self\_defer\_normalised_{i}=\frac{Approach_{i}-0}{2-0}-\frac{Self\_defer_{i}-1}{7-1}$$

$$Normative\_AA{BI}_{i}=Approac{h\_normalised}_{i}-Self\_Other\_defer\_normalised_{i}= \frac{Approach_{i}-0}{2-0}-\frac{Self\_Other\_defer_{i}-2}{14-2}$$

# Supplementary File S2: Structure of the Policy Communication Messages

The full policy framed communications are presented in the main text in Figure 1. Figure S1 below details the main manipulations in the framed communications. The central box contains the text that is consistent across the frames. Text in Boxes 1, 2 and 3 details how risk-safety frames were crossed with the altruistic frames (donor, recipient and both donor-recipient) varied across the communications.


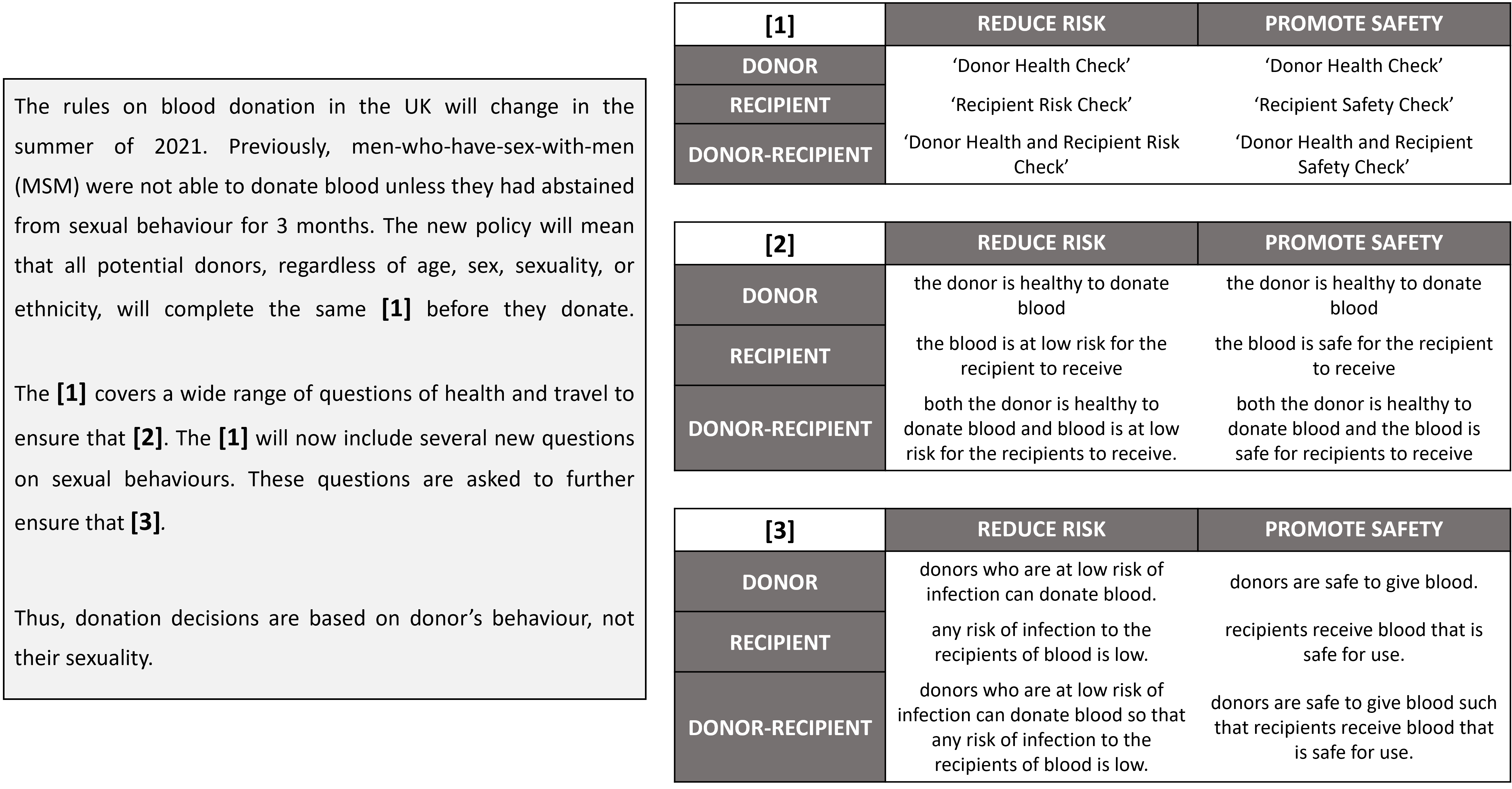


**Figure S1**: *Structure of the framed communications*

# Supplementary Files S3: Sample Structure

## **Sampling: Ethnicity and Sexuality Classifications**

Tables S1 and S2 below show the distribution of the final sample of 2,766 participants across the ONS type categorisation used to code the study data for ethnicity and sexuality. The white sample does not include white minorities (e.g., Gypsy, Roma or Irish Traveller groups or specified as ‘White other’).

**Table S1:** *Coding of ONS Ethnicity*

|  | n | Valid Percentage |
| --- | --- | --- |
|  |  |  |
| **People from Asian ethnic backgrounds** |  |  |
| Indian | 135 | 5.06 |
| Pakistani | 69 | 2.58 |
| Bangladeshi | 58 | 2.17 |
| Chinese | 80 | 3 |
| Any other Asian background, please describe | 85 | 3.18 |
|  | ***427*** |  |
| **People Black and Caribbean backgrounds** |  |  |
| African | 98 | 3.67 |
| Caribbean | 55 | 2.06 |
| Any other Black/African/Caribbean background, please describe | 22 | 0.82 |
|  | ***175*** |  |
| **People from mixed ethnic backgrounds** |  |  |
| White and Black Caribbean | 37 | 1.39 |
| White and Black African | 15 | 0.56 |
| White and Asian | 79 | 2.96 |
| Any other Mixed/Multiple ethnic backgrounds, please describe | 83 | 3.11 |
|  | ***214*** |  |
| **Other ethnic groups-** |  |  |
| Arab | 21 | 0.79 |
| Any other ethnic group, please describe | 30 | 1.12 |
|  | ***51*** |  |
| **White People** |  |  |
| English/Welsh/Scottish/Northern Irish/British | 1,598 | 59.85 |
| Irish | 18 | 0.67 |
| Gypsy or Irish Traveller | 0 |  |
| Any other White background, please describe | 177 | 6.63 |
|  | ***1793*** |  |
| Prefer not to say | 10 |  |
| Missing | 7 | 0.37 |
| **Total n** | **2,677** |  |

**Table S2:** *Coding of Sexuality*

|  | n | Valid Percentage |
| --- | --- | --- |
|  |  |  |
| **Asexual** | 50 | 1.9 |
| **Bi-Curious** | 31 | 1.2 |
| Bisexual | 366 | 14.0 |
| Gay | 118 | 4.5 |
| Heterosexual/Straight | 1,882 | 71.9 |
| Lesbian | 93 | 3.6 |
| Pansexual | 48 | 1.8 |
| Queer | 31 | 1.2 |
| Missing | 0 |  |
| Total n | **2,677** |  |

# Supplementary File S4: Secondary Outcomes

We assessed a number of secondary outcomes (“safety”, “fairness”, and “Potential Mechanism to Mitigate Non-Compliance”).

## **Perceived Safety of Blood**

The sum of two items indexes beliefs about the safety of the blood supply: (i) To what extent does this statement make you feel that blood is screened to ensure it is safe, and (ii) To what extent does the statement make you feel that the blood patients receive is safe (1 = Not at all to 7 = Completely) (*r = 0.8195, p<0.001*).

## **Perceived Fairness and Equality**

This was indexed by the sum of 4 items asking to what extent the policy communication: (i) promotes a sense of equality; (ii) promotes a sense of fairness; (iii) will encourage a wider diversity of people to donate; and (iv) suggests that donor selection feels fair (1 = Not at all, to 7 = Completely) (Cronbach’s α *=* 0.717).

## **Potential Mechanisms to Mitigate Non-Compliance**

Participants were asked to indicate the extent to which they felt asking people to use their mobile phone to aid recall over the last 3-months would be an effective strategy to increase compliance (1 = not at all, to 7 = completely).

# Supplementary File S5: Balance tests

The balance tests are presented in Tables S3a (risk frames) and S4a (other-regarding frames) for the full samples. Tables S3b (risk frames) and S4b (other-regarding frames) present corresponding tests for samples excluding the recipients of blood. There were no significant effects showing that the randomization worked and was maintained after the recipients of blood were removed.

**Table S3a:** Balance tables across risk framing manipulation (full sample)

|  | **Risk** | | **Safety** | |  |
| --- | --- | --- | --- | --- | --- |
|  | n | Mean (sd) | n | Mean (sd) | p |
| Age | 1452 | 38.29 (15.64) | 1452 | 38.61 (15.69) | 0.584 |
| Men | 1428 | 0.43 (0.49) | 1430 | 0.40 (0.49) | 0.089 |
| **Ethnicity** |  |  |  |  |  |
| Asian | 1449 | 0.16 (0.37) | 1409 | 0.15 (0.36) | 0.523 |
| Black | 1449 | 0.07 (0.26) | 1409 | 0.07 (0.25) | 0.638 |
| Mixed | 1449 | 0.07 (0.25) | 1409 | 0.06 (0.25) | 0.901 |
| White | 1449 | 0.69 (0.46) | 1409 | 0.70 (0.46) | 0.490 |
| Current donor | 1452 | 0.11 (0.32) | 1453 | 0.10 (0.30) | 0.164 |
| Blood recipient | 1444 | 0.07 (0.26) | 1443 | 0.08 (0.26) | 0.563 |
| % LGBTQ+ | 1455 | 0.29 (0.45) | 1452 | 0.30 (0.46) | 0.416 |
| % MSM | 1452 | 0.08 (0.27) | 1453 | 0.08 (0.28) | 0.417 |
| Prior  infection belief | 1439 | 20.10 (22.52) | 1445 | 19.79 (22.46) | 0.710 |
| *Note: p is the p-value of the frame manipulation coefficient in a simple linear regression of frame on the variable.* | | | | | |

**Table S3b**: Balance tables across risk framing manipulation (no recipients)

|  | **Risk** | | **Safety** | |  |
| --- | --- | --- | --- | --- | --- |
|  | n | Mean (sd) | n | Mean (sd) | p |
| Age | 1,343 | 37.74 (15.43) | 1,333 | 37.87 (15.43) | 0.821 |
| Men | 1,322 | 0.44 (0.5) | 1,313 | 0.4 (0.49) | 0.052 |
| **Ethnicity** |  |  |  |  |  |
| Asian | 1,340 | 0.17 (0.37) | 1,330 | 0.15 (0.36) | 0.306 |
| Black | 1,340 | 0.07 (0.25) | 1,330 | 0.06 (0.24) | 0.734 |
| Mixed | 1,340 | 0.08 (0.27) | 1,330 | 0.08 (0.27) | 0.954 |
| White | 1,340 | 0.67 (0.47) | 1,330 | 0.68 (0.47) | 0.572 |
| Non-donor | 1,340 | 0.65 (0.48) | 1,330 | 0.66 (0.47) | 0.637 |
| Current donor | 1,340 | 0.22 (0.41) | 1,330 | 0.23 (0.42) | 0.451 |
| Lapsed donor | 1,340 | 0.13 (0.33) | 1,330 | 0.11 (0.31) | 0.095 |
| % LGBTQ+ | 1,337 | 0.29 (0.45) | 1,333 | 0.30 (0.46) | 0.285 |
| % MSM | 1,343 | 0.08 (0.27) | 1,334 | 0.09 (0.28) | 0.494 |
| Prior  infection belief | 1,332 | 20.05 (12.63) | 1,327 | 19.70 (22.17) | 0.684 |
| *Note: p is the p-value of the frame manipulation coefficient in a simple linear regression of frame on the variable.* | | | | | |

**Table S4a**: Balance tables across other-regarding frame manipulation (full sample)

|  | **Donor** | | **Recipient** | | **Both** | |  | | |
| --- | --- | --- | --- | --- | --- | --- | --- | --- | --- |
|  | n | Mean (sd) | n | Mean (sd) | n | Mean (sd) | p |  |  |
| Age | 969 | 38.57 (15.48) | 964 | 38.57 (15.80) | 971 | 38.21 (15.72) | 0.846 | | |
| Men | 953 | 0.42 (0.49) | 949 | 0.39 (0.49) | 956 | 0.43 (0.50) | 0.183 | | |
| **Ethnicity** |  |  |  |  |  |  |  | | |
| Asian | 946 | 0.16 (0.36) | 941 | 0.15 (0.36) | 941 | 0.17 (0.37) | 0.663 | | |
| Black | 946 | 0.07 (0.26) | 941 | 0.08 (0.27) | 941 | 0.06 (0.23) | 0.240 | | |
| Mixed | 946 | 0.08 (0.27) | 941 | 0.08 (0.27) | 941 | 0.09 (0.28) | 0.787 | | |
| White | 946 | 0.69 (0.46) | 941 | 0.70 (0.46) | 941 | 0.69 (0.46) | 0.980 | | |
| Current donor | 970 | 0.11 (0.31) | 964 | 0.10 (0.30) | 971 | 0.11 (0.31) | 0.621 | | |
| Blood recipient | 967 | 0.07 (0.25) | 958 | 0.07 (0.25) | 962 | 0.08 (0.27) | 0.472 | | |
| % LGBTQ+ | 966 | 0.28 (0.45) | 960 | 0.30 (0.46) | 971 | 0.30 (0.46) | 0.606 | | |
| % MSM | 970 | 0.08 (0.27) | 964 | 0.08 (0.26) | 971 | 0.08 (0.28) | 0.774 | | |
| Prior infection belief | 961 | 20.48 (23.61) | 958 | 19.82 (21.84) | 965 | 19.54 (21.97) | 0.641 | | |
| *Note: p is the p-value of an F-test of equality across statement focus manipulation coefficients.* | | | | | | | | |  |

**Table S4b:** Balance tables across other-regarding frame manipulation (no recipients)

|  | **Donor** | | **Recipient** | | **Both** | |  | | |
| --- | --- | --- | --- | --- | --- | --- | --- | --- | --- |
|  | n | Mean (sd) | n | Mean (sd) | n | Mean (sd) | p |  |  |
| Age | 899 | 38.06 (15.30) | 893 | 27.96 (15.58) | 884 | 37.39 (15.42) | 0.617 | | |
| Men | 883 | 0.43 (0.49) | 879 | 0.4 (0.49) | 873 | 0.44 (0.5) | 0.185 | | |
| **Ethnicity** |  |  |  |  |  |  |  | | |
| Asian | 897 | 0.16 (0.36) | 890 | 0.16 (0.36) | 883 | 0.17 (0.37) | 0.748 | | |
| Black | 897 | 0.07 (0.26) | 890 | 0.07 (0.26) | 883 | 0.05 (0.23) | 0.260 | | |
| Mixed | 897 | 0.08 (0.27) | 890 | 0.08 (0.27) | 883 | 0.09 (0.28) | 0.725 | | |
| White | 897 | 0.67 (0.47) | 890 | 0.68 (0.47) | 883 | 0.66 (0.47) | 0.872 | | |
| Non-donor | 897 | 0.66 (0.47) | 890 | 0.67 (0.47) | 883 | 0.63 (0.48) | 0.187 | | |
| Current donor | 897 | 0.21 (0.41) | 890 | 0.22 (0.42) | 883 | 0.24 (0.43) | 0.167 | | |
| Lapsed donor | 897 | 0.13 (0.34) | 890 | 0.1 (0.3) | 883 | 0.12 (0.33) | 0.221 | | |
| % LGBTQ+ | 896 | 0.28 (0.45) | 890 | 0.3 (0.46) | 884 | 0.3 (0.46) | 0.533 | | |
| % MSM | 900 | 0.08 (0.28) | 893 | 0.08 (0.27) | 884 | 0.08 (0.28) | 0.920 | | |
| Prior infection belief | 891 | 20.44 (23.58) | 889 | 19.71 (21.65) | 879 | 19.78 (21.92) | 0.639 | | |
| *Note: p is the p-value of an F-test of equality across statement focus manipulation coefficients.* | | | | | | | | |  |

# Supplementary File S6: Other Regarding Focused Frames Manipulation Checks

In terms of focus (Who is the focus of the statement the donor (0), both equally (5), and the patient (10)). Across all treatments, participants. Om average, reported the statement tended towards a focus on the donor and recipient equally (*M =* 3.42, *CI = [3.33, 3.51])* (Table S5).

In terms of salience (To what extent does the statement make you think about the patients who receive blood? (1 = Not at all to 7 = Completely)), participants reported thinking about the recipient more in the combined donor-recipient condition (*M =* 4.76*,* $CI_{95\%}$=[4.65, 4.86]) than to the donor only (*M =* 4.58*,* $CI_{95\%}$=[4.47, 6.69]*, p =* 0.021) and the recipient only condition (*M =* 4.56, $CI_{95\%}$=[4.44, 4.67]*, p =* 0.012) (Table S5). Compared to the mid-point of 3.5 overall participants tended to think more about the patients (*M =* 4.63*,* $CI_{95\%}$=[4.57, 4.69]) t_one-sample_ = 37.73; p < .0001). The extent of thinking about the recipient was not significantly different compared to the mid-point (3.5) for the “donor” frame (t_one-sample_ = -0.952; p = .341) or the “recipient” frame (t_one-sample_ = -1.250; p = .212) but was for the “donor and recipient” frame (t_one-sample_ = 2.346; p = .019).

Thus, the other-regarding frames, especially combined donor-recipient frame, afford thoughts about the recipient and a general focus close to both ‘donor and recipient’.

**Table S5:** Focus and Salience manipulation checks

|  |  | Mean | CI [95%] |  | p |
| --- | --- | --- | --- | --- | --- |
| **Focus (0 Donor to 10 Recipient)** | |  |  |  |  |
|  | **Overall** | **3.42** | **3.33, 3.51** |  |  |
|  | 1. Donor | 3.37 | 3.21, 3.42 | 1 v 2 | 0.843 |
|  | 2. Recipient | 3.40 | 3.23, 3.56 | 1 v 3 | 0.317 |
|  | 3. Both | 3.49 | 3.33, 3.65 | 2 v 3 | 0.423 |
| **Salience of the recipient** | |  |  |  |  |
|  | **Overall** | **4.63** | **4.57, 4.69** |  |  |
|  | 1. Donor | 4.58 | 4.47, 4.69 | 1 v 2 | 0.809 |
|  | 2. Recipient | 4.56 | 4.44, 4.67 | **1 v 3** | **0.021** |
|  | 3. Both | 4.76 | 4.65, 4.86 | **2 v 3** | **0.012** |

*Note. Focus is* “Who is the focus of the statement the donor (0), both equally (5), and the patient (10),”*, Salience of the recipient is* “To what extent does the statement make you think about the patient who receive blood?” (1 = Not at all to 7 = Completely). P values from independent t-tests.

# Supplementary File S7: Negative Suppressor Effect of Age on the LGBTQ+ - Approach Association

A negative suppressor effect occurs when the inclusion of a suppressor variable (S) in a regression equation causes a reversal of sign for an estimated parameter between variables X and Y [1-3]. That is, if the bivariate association between X and Y is positive, but this reverses and becomes negative when S is included in the equation S is the suppressor variable.

In Table 3 in the main text, we have a suppressor effect on the relationship between LBBTQ+ status (LGBTQ+ or Straight) and approach (intention-to-donate). The bivariate association between LBBTQ+ status and approach is positive as LGBTQ+ people endorse a higher level of approach (*M* = 0.73, *Sd* =0.32, *n* = 851) than straight people (*M* = 0.70, *Sd* =0.35, *n* = 2,045) (t _(2894)_ = -2.35, p = .0186). This is reversed in the regression equation in Table 3. Entering the other variables sequentially showed that it was the inclusion of age that caused this reversal. As such, we considered age as the suppressor variable. To interpret how age is acting as a suppressor we examined its association with approach and LGBTQ+ status. The association of age with approach was negative (*r* = -.23, p < .0001), such that younger people are less likely to approach (intend-to-donate). The association of age with LGBTQ+ status was also negative: LGBTQ+ people were younger (*M* = 31.24, *Sd* =11.43, *n* = 851) than straight people (*M* = 41.44, *Sd* =16.20, *n* = 2,045) (t _(2894)_ = 16.72, p < .0001). Thus, younger people, who are more likely to be from the LGBTQ+ community, approach less. Therefore, age captures any overlapping variance between LGBTQ+ status and approach suppressing the positive zero-order effect and reversing it. Thus, people from LGBTQ+ communities are more likely to intend to donate than straight people, and this basic effect is suppressed in the presence of age.

**References**

[1]. Paulhus DL, Robins RW, Trzesniewski KH, Tracy, JL. Two replicable suppressor situations in personality research. Multivar Behav Res, 2004; 39: 303–328.

[2]. Conger AJ. A revised definition for suppressor variables: A guide to their identification and interpretation. Educ Psychol Meas 1974; 34: 35–46.

[3]. Martinez Gutierrez, N, Cribbie, R. (). Incidence and interpretation of statistical suppression in psychological research. Can J Behav Sci, 2021; 53: 480–488.

# Supplementary File S8: Margins Analysis of Approach-Avoidance Interactions

The margin analyses for the interactions observed in Table 3 in the main text are presented in Tables S6a and S6b and Figure S2 below. These show that the combination of a risk-frame with the donor-focused frame results in higher levels of feeling deterred from donating for self, others and normative considerations.

Figure S2 show that the highest levels of feeling deterred from donating are observed when the donor-focus and risk-frames are combined. In support of this, the margin analysis reported in Table S6a shows that, compared to the donor-focus risk-frame combination, reported levels of feeling deterred from donating blood are lower when the recipient-focused frame is combined with the risk-frame. Furthermore, the margin analysis reported in Table S6b shows that, compared to the risk-frame combined with a donor-focused frame, reported levels of feeling deterred from donating blood, are lower when the donor-focused frame is combined with a safety-frame.

**Table S6a.** Margins for Risk Frame by Other Regarding Frames Interactions

|  | Delta-method |  |  |  | Delta-method |  |  |  | Delta-method |  |  |  |
| --- | --- | --- | --- | --- | --- | --- | --- | --- | --- | --- | --- | --- |
|  | Deterred: Self | | | | Deterred: Other | | | | Deterred: Normative | | | |
|  | dy/dx  (std. err.) | P= | 95% interval | | dy/dx (std. err.) | P= | 95% interval | | dy/dx (std. err.) | P= | 95% interval | |
|  |  |  | Lower | Upper |  |  | Lower | Upper |  |  | Lower | Upper |
| **Donor Frame** (Comparison) |  |  |  |  |  |  |  |  |  |  |  |  |
| **Recipient Frame** |  |  |  |  |  |  |  |  |  |  |  |  |
| **Risk Frame** |  |  |  |  |  |  |  |  |  |  |  |  |
| Safety | 0.0107  (0.0162) | .508 | -0.0210 | 0.0424 | 0.0327  (0.0179) | .069 | -0.0025 | 0.0679 | 0.0216  (0 .0147) | .143 | -0.0073 | 0.0504 |
| Risk | **-0.0367**  **(0.0158)** | **.020** | **-0.0677** | **-0.0057** | **-0.0351**  **(0.0176)** | **.046** | **-0.0696** | **-0.0006** | **-0.0360**  **(0.0144)** | **.012** | **-0.0643** | **-0.0078** |
| **Both Donor and Recipient Frame** |  |  |  |  |  |  |  |  |  |  |  |  |
| **Risk Frame** |  |  |  |  |  |  |  |  |  |  |  |  |
| Safety | 0.01278  (0.01601) | .425 | -0.0186 | 0.0442 | 0.0245  (0.0178) | .168 | -0.0104 | 0.0594 | 0.0185  (0.0146) | .204 | -0.0100 | 0.0471 |
| Risk | -0.0208  (0.0160) | .195 | -0.0522 | 0.0107 | -0.0248  (0.0178) | .164 | -0.0598 | 0.0101 | -0.0228  (0.0146) | .119 | -0.0514 | 0.0058 |

**Table S6b.** Margins for Risk Frame by Other Regarding Frames Interactions

|  | Delta-method |  |  |  | Delta-method |  |  |  | Delta-method |  |  |  |
| --- | --- | --- | --- | --- | --- | --- | --- | --- | --- | --- | --- | --- |
|  | Deterred: Self | | | | Deterred: Other | | | | Deterred: Normative | | | |
|  | dy/dx  (std. err.) | P= | 95% interval | | dy/dx (std. err.) | P= | 95% interval | | dy/dx (std. err.) | P= | 95% interval | |
|  |  |  | Lower | Upper |  |  | Lower | Upper |  |  | Lower | Upper |
| **Risk Frame** (Comparison) |  |  |  |  |  |  |  |  |  |  |  |  |
| **Safety Frame** |  |  |  |  |  |  |  |  |  |  |  |  |
|  |  |  |  |  |  |  |  |  |  |  |  |  |
| Donor | **-0.0437 (0.0160)** | **.006** | **-0.0750** | **-0.0123** | **-0.0483 (0.0177)** | **.007** | **-.00831** | **-0.0135** | **-0.0458**  **(0.0145)** | **.002** | -0.0744 | -0.0173 |
| Recipient | 0.0037 (0.0160) | .816 | -0.0276 | 0.0351 | 0.0195  (0.0178) | .272 | -0.0153 | .05439 | 0.0117  (0.0146) | .421 | -0.0168 | 0.0403 |
| Both | -0.0101  (0.0161) | **.**529 | -0.0416 | 0.0214 | 0.0011  (0.0178) | .952 | -0.0340 | 0.0361 | -0.0045  (0.0146) | .757 | -0.0332 | 0.0241 |


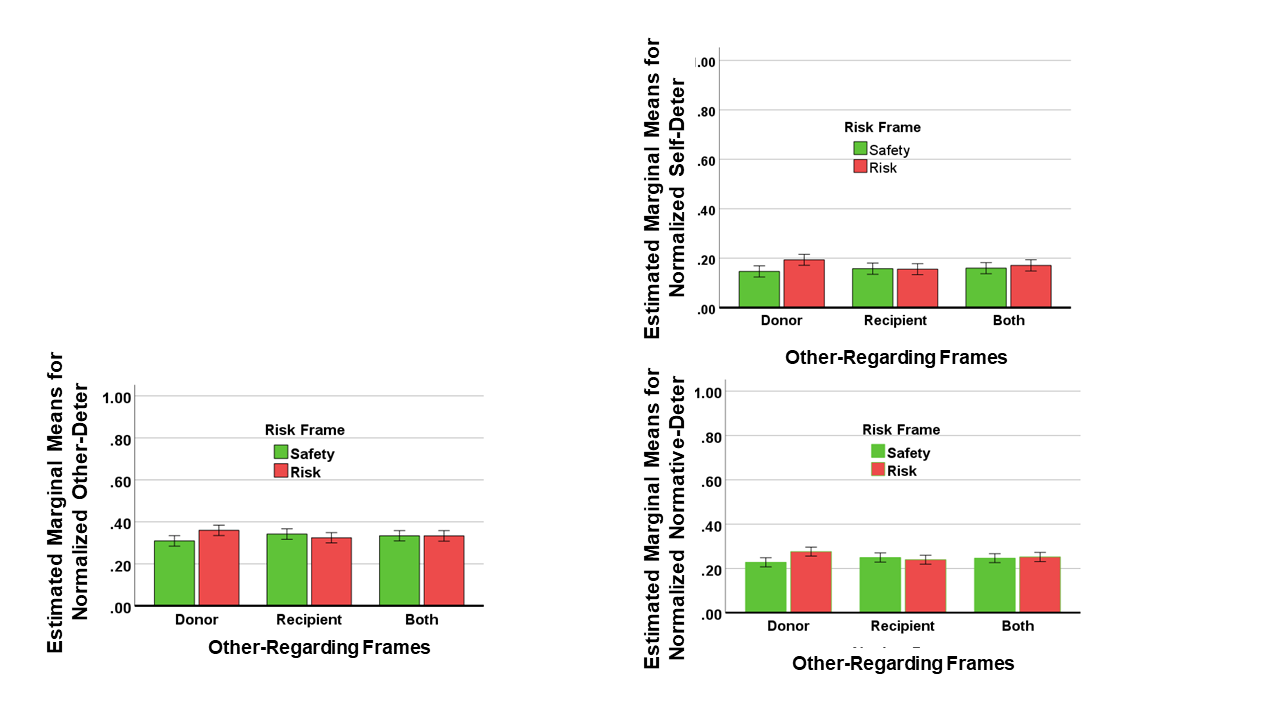


**Figure S2**: The Interaction of Risk Frames by Other-Regarding Frames for Reported Levels of being Deterred from Donating Blood .

Error Bars are 95% C.I.s

# Supplementary File S9: Regression Models for Awareness of Mechanisms Linked to the Anticipation of Under-Reporting of Sexual Behaviour

Table S7 details the OLS regression analyses for the predictors of the three anticipated mechanisms associated with the under-reporting of sexual behavior.

**Table S7**: OLS regressions for Awareness of mechanisms leading to under-reporting of sexual behaviors

|  | **(1)** | | | **(2)** | | | | | **(3)** | | | | |  |  |
| --- | --- | --- | --- | --- | --- | --- | --- | --- | --- | --- | --- | --- | --- | --- | --- |
|  | **Forgetting** | | | **Negative Emotions**  **(Shame and Embarrassment)** | | | | | **Irrelevance**  **(Blood is Tested)** | | | | |  |  |
|  | β | | CI [95%] | | β | | | CI [95%] | | β | | | CI [95%] | | |
| **Risk Frame** |  | |  | |  | | |  | |  | | |  | | |
| Safety | 0.02 | | [-0.189,0.229] | | -0.024 | | | [-0.188,0.141] | | 0.106 | | | [-0.104,0.315] | | |
| **Other-Regarding Frame** |  | |  | |  | | |  | |  | | |  | | |
| Recipient | 0.027 | | [-0.180,0.235] | | 0.105 | | | [-0.058,0.268] | | 0.054 | | | [-0.153,0.261] | | |
| Both | 0.069 | | [-0.141,0.279] | | 0.056 | | | [-0.108,0.221] | | -0.032 | | | [-0.242,0.179] | | |
| **Risk X Other-Regarding Frames** |  | |  | |  | | |  | |  | | |  | | |
| Safety x Recipient | -0.05 | | [-0.346,0.246] | | 0.035 | | | [-0.197,0.267] | | -0.107 | | | [-0.403,0.189] | | |
| Safety x Both | -0.091 | | [-0.388,0.206] | | 0.018 | | | [-0.215,0.251] | | 0.027 | | | [-0.270,0.324] | | |
|  |  | |  | |  | | |  | |  | | |  | | |
| Age | **-0.010***** | | [-0.015,-0.005] | | **-0.005**** | | | [-0.009,-0.002] | | 0.002 | | | [-0.003,0.007] | | |
| Men | **-0.300***** | | [-0.425,-0.176] | | **-0.246***** | | | [-0.344,-0.149] | | **-0.443***** | | | [-0.568,-0.319] | | |
| LGBQ+ | 0.069 | | [-0.081,0.218] | | -0.104 | | | [-0.222,0.013] | | -0.081 | | | [-0.231,0.068] | | |
| **Ethnicity** |  | |  | |  | | |  | |  | | |  | | |
| Asian | **0.348***** | | [0.167,0.529] | | 0.075 | | | [-0.067,0.217] | | 0.123 | | | [-0.058,0.304] | | |
| Black | **0.322*** | | [0.067,0.576] | | **0.328**** | | | [0.128,0.529] | | **0.303*** | | | [0.048,0.558] | | |
| Mixed | 0.148 | | [-0.083,0.380] | | 0.174 | | | [-0.007,0.356] | | 0.074 | | | [-0.158,0.305] | | |
| **Donor status** |  | |  | |  | | |  | |  | | |  | | |
| Lapsed donor | -0.131 | | [-0.289,0.026] | | 0.031 | | | [-0.093,0.154] | | -0.097 | | | [-0.255,0.060] | | |
| **Current donor** | **-0.129** | | **[-0.321,0.062]** | | **-0.190*** | | | [-0.341,-0.040] | | -0.148 | | | [-0.340,0.043] | | |
| Constant | 4.434*** | | [4.165,4.703] | | 5.611*** | | | [5.400,5.822] | | 5.064*** | | | [4.796,5.333] | | |
| $R^{2}$ | 0.040 |  | | | | 0.029 |  | | | | 0.026 |  | | | |
| N | 2552 |  | | | | 2548 |  | | | | 2551 |  | | | |
| ***Note****: * p < 0.05, ** p < 0.01, *** p < 0.001.: Frames: Risk frame is the comparison condition. Other-Regarding Frames, the donor frame is the comparison condition. Ethnicity = people from white ethnicity backgrounds act as the comparison community, Donor status = non-donors are the comparison group. Coefficients are unstandardized.* | | | | | | | | | | | | | | |  |

# Supplementary File S10: Safety and Fairness Regression Models

There were no significant framing effects on the perceived safety to the blood supply or equality and fairness of the policy (Table S8). However, there were demographic effects. Older respondents ($\beta_{safety}=$-0.020, ${CI}_{safety}=$ [-0.027,-0.012], $\beta_{fair}=$-0.013, ${CI}_{fair}=$ [-0.023,-0.003]) and respondents from Asian ( $\beta_{safety}=$-0.821, ${CI}_{safety}=$ [-1.122,-0.519], $\beta_{fair}=$-0.963, ${CI}_{fair}=$ [-1.343,-0.584]), Black ($\beta_{safety}=$-0.740, ${CI}_{safety}=$ [-1.165,-0.315]), and Mixed ($\beta_{safety}=$-0.398, ${CI}_{safety}=$ [-0.784,-0.012], $\beta_{fair}=$-0.733, ${CI}_{fair}=$ [-1.218,-0.247]) ethnic backgrounds, relative to white respondents, reported lower perceived safety and fairness. Lapsed donors ($\beta_{safety}=$0.576, ${CI}_{safety}=$ [0.313, 0.839]) and current donors ($\beta_{safety}=$0.941, ${CI}_{safety}=$ [0.622, 1.261]) reported higher perceived safety scores than non-donors. Also, LGBQ+ respondents reported greater perceptions of safety than straight identifying respondents ($\beta_{safety}=$0.656, ${CI}_{safety}=$ [0.407,0.906]). Lapsed donors reported higher perceived fairness than non-donors ($\beta_{fair}=$0.502, ${CI}_{fair}=$ [0.172,0.833]). Men, compared to women, reported lower levels of perceived safety ($\beta_{safety}=$-0.212, ${CI}_{safety}=$ [-0.420,-0.004]), and fairness ($\beta_{fair}=$-0.666, ${CI}_{fair}=$ [-0.927,-0.405]).

**Table S8**: OLS regressions on perceived safety and fairness/equality.

|  | **(1)** | | **(2)** | |
| --- | --- | --- | --- | --- |
|  | **Perceived safety** | | **Fairness and Equality** | |
|  | β | CI [95%] | β | CI [95%] |
| **Risk Frame** |  |  |  |  |
| Safety | 0.038 | [-0.311,0.387] | 0.092 | [-0.347,0.531] |
| **Other-Regarding Frames** |  |  |  |  |
| Recipient | -0.141 | [-0.486,0.205] | -0.15 | [-0.585,0.284] |
| Both | -0.144 | [-0.495,0.206] | -0.106 | [-0.547,0.334] |
| **Risk X Other-Regarding Frames** |  |  |  |  |
| Safety x Recipient | -0.041 | [-0.535,0.452] | 0.044 | [-0.577,0.665] |
| Safety x Both | -0.091 | [-0.586,0.405] | 0.003 | [-0.620,0.626] |
|  |  |  |  |  |
| *Controls* |  |  |  |  |
| Age | **-0.020***** | [-0.027,-0.012] | **-0.013*** | [-0.023,-0.003] |
| Men | **-0.212*** | [-0.420,-0.004] | **-0.666***** | [-0.927,-0.405] |
| LGBQ+ | **0.656***** | [0.407,0.906] | 0.084 | [-0.230,0.398] |
| **Ethnicity** |  |  |  |  |
| Asian | **-0.821***** | [-1.122,-0.519] | **-0.963***** | [-1.343,-0.584] |
| Black | **-0.740***** | [-1.165,-0.315] | -0.181 | [-0.715,0.354] |
| Mixed | **-0.398*** | [-0.784,-0.012] | **-0.733**** | [-1.218,-0.247] |
| **Donor status** |  |  |  |  |
| Lapsed donor | **0.576***** | [0.313,0.839] | **0.502**** | [0.172,0.833] |
| Current donor | **0.941***** | [0.622,1.261] | 0.262 | [-0.140,0.664] |
|  |  |  |  |  |
| Constant | 11.921*** | [11.473,12.368] | 25.148*** | [24.584,25.711] |
| $R^{2}$ | 0.065 |  | 0.029 |  |
| N | 2551 |  | 2553 |  |
| ***Note****: * p < 0.05, ** p < 0.01, *** p < 0.001.: Frames: Risk frame is the comparison condition. Other-Regarding Frames the donor frame is the comparison condition. Ethnicity = people from white ethnicity backgrounds act as the comparison community, Donor status = non-donors are the comparison group.* | | | | |
